# Bragg-Berry flat reflectors for transparent computer-generated holograms and waveguide holography with visible color playback capability

**DOI:** 10.1038/s41598-020-65102-0

**Published:** 2020-05-18

**Authors:** Seong Yong Cho, Masaru Ono, Hiroyuki Yoshida, Masanori Ozaki

**Affiliations:** 1 Division of Electrical, Electronic and Information Engineering, Osaka University, 2-1 Yamadaoka, Suita Osaka, 565-0871 Japan; 2 Precursory Research for Embryonic Science and Technology (PRESTO), Japan Science and Technology Agency (JST), 4-1-8 Honcho, Kawaguchi Saitama, 332-0012 Japan

**Keywords:** Applied optics, Optical materials and structures, Liquid crystals

## Abstract

Various approaches are being pursued to realize compact optical elements with the ability to manipulate light, but it is difficult to simultaneously achieve high reflectivity and the ability to see through the element. Here, we present a reflective computer-generated hologram that is completely transparent in the visible, based on the Berry (geometric) phase in a self-organizing Bragg reflector. The Bragg reflector has a helical dielectric tensor distribution with the phase information imprinted in the distribution of the optic axis on the substrate. The structure possesses only a single Fourier component and high-order reflections are suppressed; thus, the device appears completely transparent by setting the main reflection band outside the visible range for all angles of incidence accessible by ambient light. On the other hand, the encoded phase information can be played back using visible light by increasing the accessible incidence angle, which we demonstrate experimentally by (i) attaching a coupling prism, and (ii) integrating the device in a waveguide. Bragg-Berry reflectors thus enable a new route to realize advanced optical elements with no apparent reflection in the visible region.

## Introduction

Conventional optical systems use bulky lenses and mirrors to control light propagation, but the spread of wearable devices is pushing the need for compact optical elements with the capability to control light^1^. One of the most intensively pursued technologies is the metasurface, in which sub-wavelength structures are used to control the amplitude, phase, and polarization of impinging light^2,3^. For a given material and wavelength, arbitrary complex transmittance can be realized by nanostructure design; thus, various, and often numerous functions can be integrated in an ultra-small device by designing the distribution of nanostructures. In particular, algorithms developed for computer-generated holography (CGH) can be employed to numerically obtain the complex transmittance distribution required to achieve a particular optical functionality^4,5^. Metasurface deflectors^2,6,7^, lenses^6–11^, holograms^11–16^, and beam shapers^17^, both operating in infrared (IR) and visible wavelengths, have been demonstrated.

Another approach to control light is to use the geometric phase or Pancharatnam-Berry (PB) phase in anisotropic media^18,19^. When circularly polarized (CP) light propagates through an anisotropic medium with half-wave retardation, the light flips its polarization handedness as well as acquires a phase that is proportional to twice the azimuthal orientation of the optic axis^20–28^. Thus, various optical functions can be realized by appropriately designing the optic axis distribution. Liquid crystals (LCs) are particularly attractive materials to realize devices based on the PB phase effect^21–28^, since they possess the potential for the fabrication of large-area devices by coating the materials on a substrate with appropriate patterning. Deflectors^22–24^, phase plates for vector coronagraphy^25^, and holograms^26–28^ have been reported as well as lens arrays as large as 50 mm^28^.

Despite the enormous development in novel diffractive optical elements, it is still a challenge to achieve high reflectivity and the capability to see through the element. The capability of seeing through an element is particularly useful for various applications where virtual information is displayed on transparent screen such as window or wall-type display. However, metasurfaces and PB phase elements made in reflection mode use an opaque substrate^10–13^, which eliminates the ability to see through the element. In addition, standard holographic optical elements (HOEs) are not completely transparent due to the appearance of Bragg reflection in the visible light region^1^. It is not a trivial task to achieve a HOE with both high reflectance and the property of being able to see through the device.

In this paper, we present a reflective CGH that is completely transparent in visible region, but is capable of playing back the encoded information by visible light. The device uses a cholesteric liquid crystal (ChLC), which from an optical perspective is a periodic anisotropic structure with a helical modulation in the optic axis^29^. ChLCs have been long known as self-organizing Bragg reflectors, but was recently shown that the phase of Bragg reflected light can be controlled by modifying the structural phase of ChLCs, in a similar manner to the PB phase^30–38^. For light impinging from normalcy on the structure, the reflected optical phase varies in proportion to the spatial phase of the helical structure (helix phase), allowing 0–2π control of optical phase by a 0-π variation in the helix phase. Based on the concept, various reflection-type HOEs including beam deflectors^30,36,37^, polychromatic vortex beam generators^32,33,38^, wide-angle light diffusers^34^, and double-sided holograms^35^ have been demonstrated by appropriately designing the helical phase distribution of ChLCs on a substrate. The unique properties such as self-organization and easy control of operation bandwidth make ChLCs as attractive materials than existing conventional HOEs. ChLCs thus constitute a new class of self-organizing HOEs, which are sometimes referred to as Bragg-Berry (BB) optical elements^38^. We exploit this feature as well as the fact that ChLCs show insignificant high-order reflections to realize the transparent, reflective CGH.

## Results

**Figure 1:**
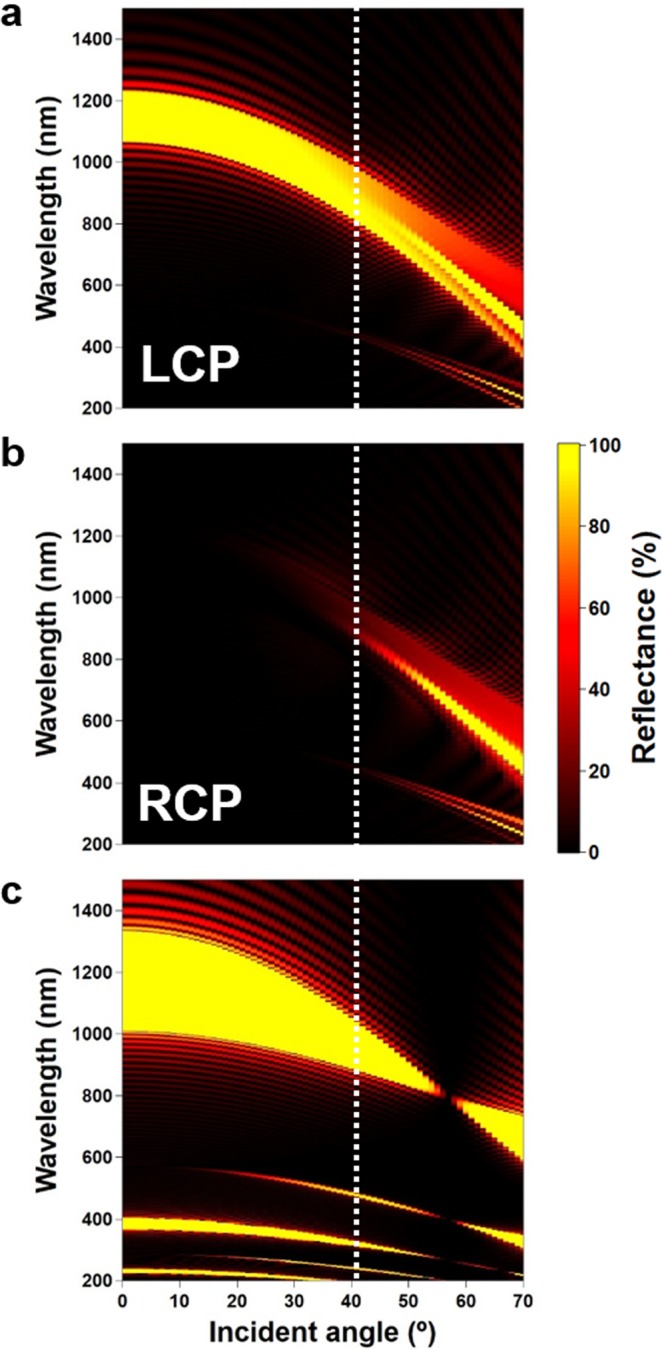
Simulated reflection spectra of a ChLC (anisotropic helical structure) and a standard quarter-wave plate. (**a**,**b**) Incident angle dependence of the reflection spectra for a ChLC upon LCP and RCP light incidence. (**c**) Reflection spectra of a quarter-wave stack upon LP light incidence. The white dashed lines show the maximum accessible incident angle for the device placed in air.

ChLCs are formed by rod-like LC molecules and have a helical structure. Taking the helical axis as the z-axis, the dielectric tensor distribution in a ChLC is expressed as follows:

1 $${\boldsymbol{\varepsilon }}(z)=\,(\begin{array}{cc}\bar{\varepsilon } & 0\\ 0 & \bar{\varepsilon }\end{array})+\alpha (\begin{array}{cc}\cos \,2(qz+\varphi ) & \sin \,2(qz+\varphi )\\ \sin \,2(qz+\varphi ) & -\cos \,2(qz+\varphi )\end{array})$$

where $\bar{\varepsilon }=({n}_{e}^{2}+{n}_{o}^{2})/2$, $\alpha =({n}_{e}^{2}-{n}_{o}^{2})/2$, $q=2{\rm{\pi }}/p$, *φ* is the helix phase, *n*_o_ and *n*_e_ are the ordinary and extraordinary refractive indices and *p* is the helical pitch. For light propagating along the helix, the helical modulation in the dielectric tensor leads to Bragg reflection of CP light over a wavelength band given by *n*_o_*p* − *n*_e_*p*. Inspection of Eq. 1 shows that only a single Fourier component exists, which means that all high-order reflections are suppressed^39^. Complete mitigation occurs only for normal incidence, but even for oblique incidence, it is much smaller compared to a standard quarter-wave stack made from a regular stack of two dielectric materials with different refractive indices and thickness. In Fig. 1, we compare the incident angle dependence of reflection band of a ChLC (with left-handed helicity) and a standard quarter-wave stack, calculated by the 4 × 4 matrix method^40^. In the calculation, the incident angle is defined as the angle between the propagation direction of light in the glass substrate measured from the boundary normal.

From Fig. 1a,b, we see that high-order reflections are indeed absent for normal incidence in the ChLC, and that second-order reflection increases only slightly with the incident angle. When the device is in air, the maximum incident angle from the glass substrate to the ChLC is limited to 41° (assuming a refractive index of 1.53 for the glass substrate); at this angle, the maximum reflectance is approximately 10% for both CPs with a narrow bandwidth of <5 nm, which is comparable to the interference fringes originating from the sandwiched optical structure. The ChLC device is thus rendered effectively transparent under ambient lighting. The quarter-wave stack, on the other hand, shows odd-order reflections at normal incidence, with the even-order reflections appearing upon oblique incidence (at approximately *λ*_center_/*m*, where *λ*_center_ and *m* are the central wavelength of Bragg reflection for normal incidence and the reflection order, respectively). The second-order reflectance in the visible light region exceeds 90% at 41° incidence, as shown in Fig. 1c.

**Figure 2:**
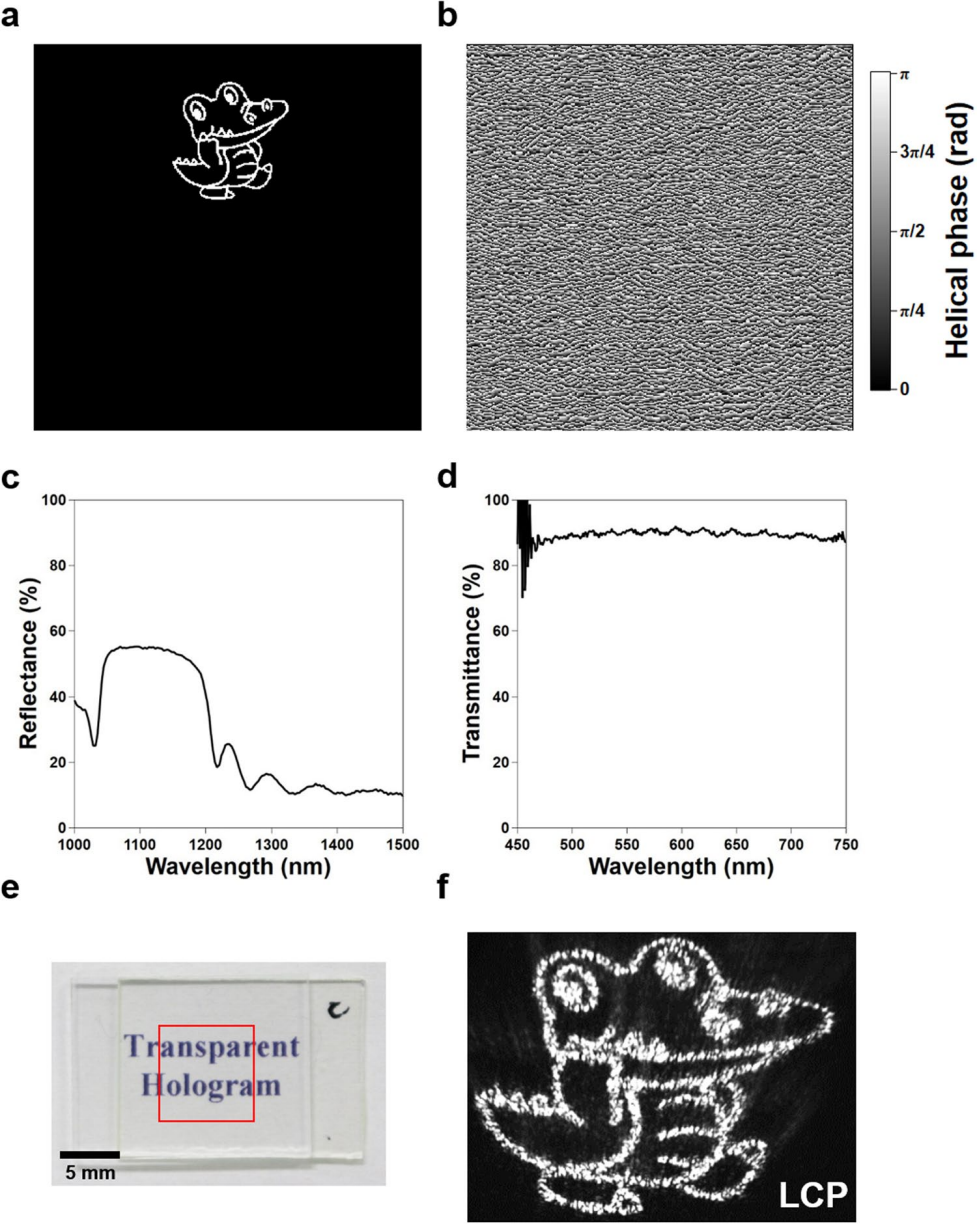
Design and reconstruction of the BB hologram for normal incidence. (**a**) Off-axis source image used: the OSAKA UNIVERSITY mascot Dr. Wani. (**b**) Helix phase distribution corresponding to the source image calculated by the G-S algorithm. (**c**) The reflection spectrum of the BB hologram in the infrared light region for LP light incidence. (**d**) The transmission spectrum of the fabricated BB hologram in the visible light region. (**e**) Photograph of the device, where the red square indicates the photo-patterned region with size of ~8 × 8 mm^2^. (**f**) Hologram reconstructed using a laser with wavelength of 1100 ± 5 nm upon normal incidence.

A CGH can be implemented in the transparent ChLC by appropriately designing its helix phase. Figure 2a shows the design of the CGH (Osaka University mascot Dr. Wani) we implemented in experiment. The optical phase distribution was calculated by the Gerchberg-Saxton (G-S) algorithm^4,5^, and converted to the helix phase distribution as shown in Fig. 2b by multiplying a factor of 0.5 (see Supplement 1 Section S1 for the phase retrieval algorithm). The retrieved phase distribution was patterned through a maskless photoalignment technology^30^ on a sandwiched cell with a gap width of 9 µm. The phase step of the patterning process was 10° (18 phase levels). The CGH comprised 512 × 384 pixels with a pixel size of 2.7 × 2.7 µm^2^, which gave a patterned area of 1.3 × 1.0 mm^2^. The patterning was performed over a 6 × 8 array, giving a device with a total area of ~ 8 × 8 mm^2^. A left-handed ChLC with reflection band between approximately 1050 nm and 1200 nm for normal incidence (Fig. 2c) was injected in the cell after the patterning process. Figure 2d shows the transmission spectrum of the BB hologram in the visible range, which, in accordance with simulations, shows no high-order reflections and gives a high average transmittance of 90% (note that losses are due to the fact that there are no antireflection coatings on the device). The photograph in Fig. 2e demonstrates that the hologram device is transparent under ambient lighting, i.e., non-polarized light, despite the patterning of the helix phase.

The encoded phase information in the transparent hologram was played back using a super-continuum light source (NKT Photonics, SuperK COMPACT) with a band-pass filter (central wavelength of 1100 nm with bandwidth of 10 nm). Figure 2f shows the reconstructed hologram observed upon left circularly polarized (LCP) illumination. For normal incidence, the BB hologram shows strong CP selectivity, and reconstructs only LCP light. The conversion efficiency of the hologram is defined as the intensity ratio of the diffracted light (without the 0^th^ order intensity) to the incident intensity^35^, and the measured value was 76% for LCP incidence. The reflectance was approximately 83%, which gives a diffracted-to-zero order light efficiency of 92%. We believe that losses are attributed to manufacturing imperfections, but the efficiency is quite high because of the nature of LCs to change their orientation continuously, even on substrates with a discrete change in orientational easy axis.

**Figure 3:**
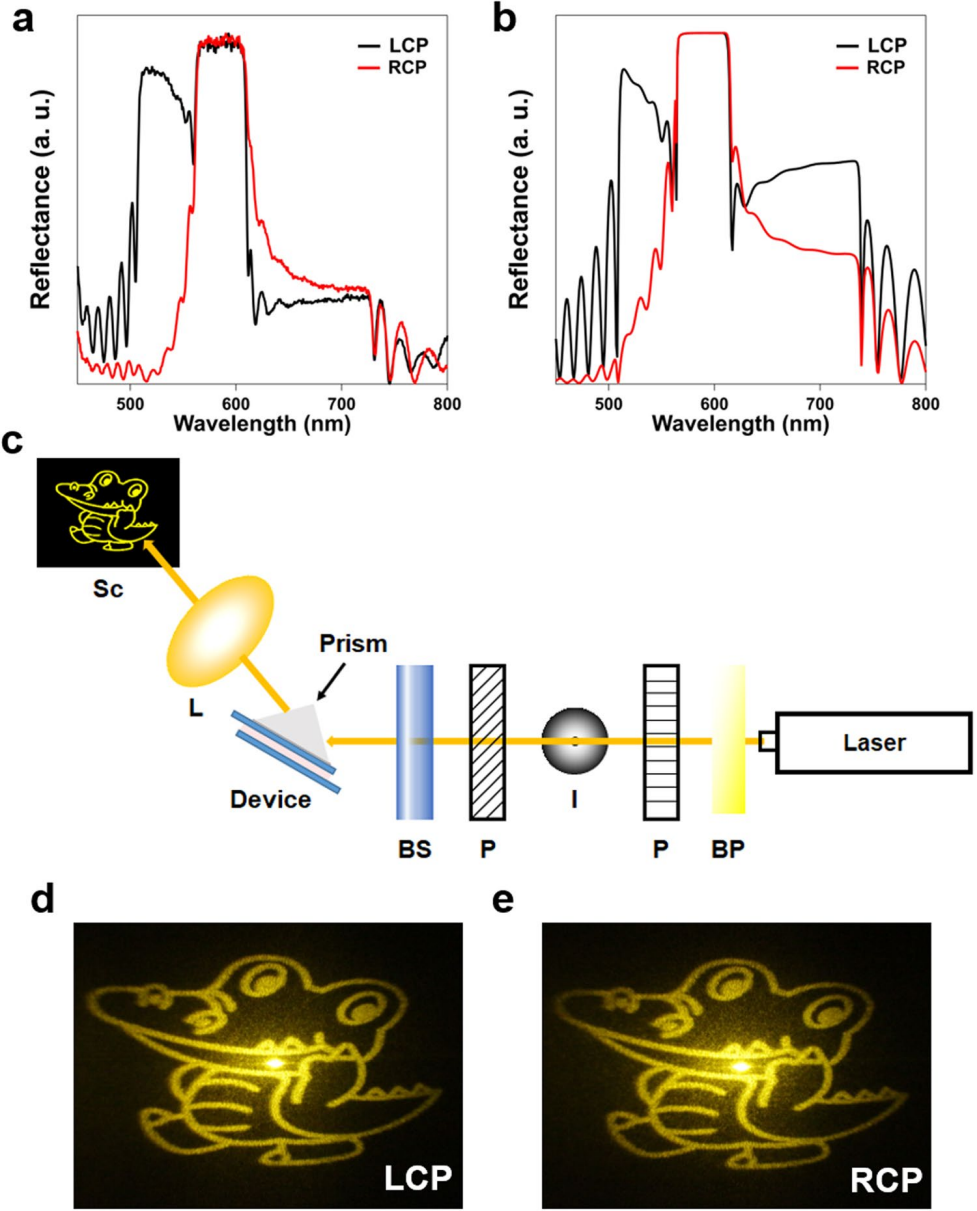
Visible playback from the transparent hologram by oblique light incidence. (**a**,**b**) Experimental (**a**) and simulated (**b**) reflectance spectra of the BB hologram for LCP and RCP illumination at an incident angle of 61°. (**c**) Schematic of the laser set up. BP: band-pass filter; P: polarizer; I: iris; BS: Babinet-Soleil compensator; L: lens; Sc: screen. The figure was drawn using Microsoft PowerPoint 2016. (**d**,**e**) Observed holographic images for LCP (**d**) and RCP (**e**) light illumination using a laser with wavelength of 580 ± 5 nm.

As shown in Fig. 2e, the BB hologram appears transparent under ambient lighting due to the limited angles of incidence on the device. We now demonstrate that the hologram can be played back using visible light by increasing the incident angle, the simplest method of which is to use an index-matching prism. A BK-7 prism was attached on the device to increase the accessible angles of light (see Supplement 1 Section S2 for the extension of accessible angles). For visible playback of encoded phase information, we choose 61° as the incident angle in the glass substrate, for which the experimental and simulated reflection spectra are plotted in Fig. 3a,b, respectively. For large angles of incidence, ChLCs exhibit a reflection band that is independent on the polarization (see Supplement 1 Section S3 and S4 for the experimental angular dependence of reflectance in the visible light region and a description of the physical mechanism of polarization-independent reflection)^41^. This is convenient since this enables efficient playback of the hologram irrespective of the incident polarization. The total reflection band appears around 580 nm with a bandwidth of approximately 40 nm, which is in good agreement with theory.

The hologram was reconstructed by inserting a band-pass filter (central wavelength of 580 nm with bandwidth of 10 nm) in the super-continuum light source using the laser set-up shown in Fig. 3c. The handedness of the incident CP light was controlled using a polarizer and a Babinet-Soleil compensator, and the angle of the BB hologram device was set to 27° using a rotatable stage to achieve the target incident angle (see Supplement 1 Section S2). Figure 3d,e show the holographic images for LCP and right circularly polarized (RCP) light projected on a screen (placed at 30 cm away from the device) through a lens (*f* = 150 mm). Note that for this experiment, the source image was centered around the zero-order spot and enlarged by 3 times to obtain a larger image (see Supplement 1 Section S5 for the source image and helical phase distribution for oblique incidence). Upon illumination of either CP, the encoded hologram is clearly observed in a yellow color (λ = 580 nm), demonstrating the capability of playback by visible light. Supplement Movie S1 shows two identical transparent holograms illuminated obliquely by a red laser (λ = 650 nm) with linear polarization, where only the sample with the prism generates a hologram image.

**Figure 4:**
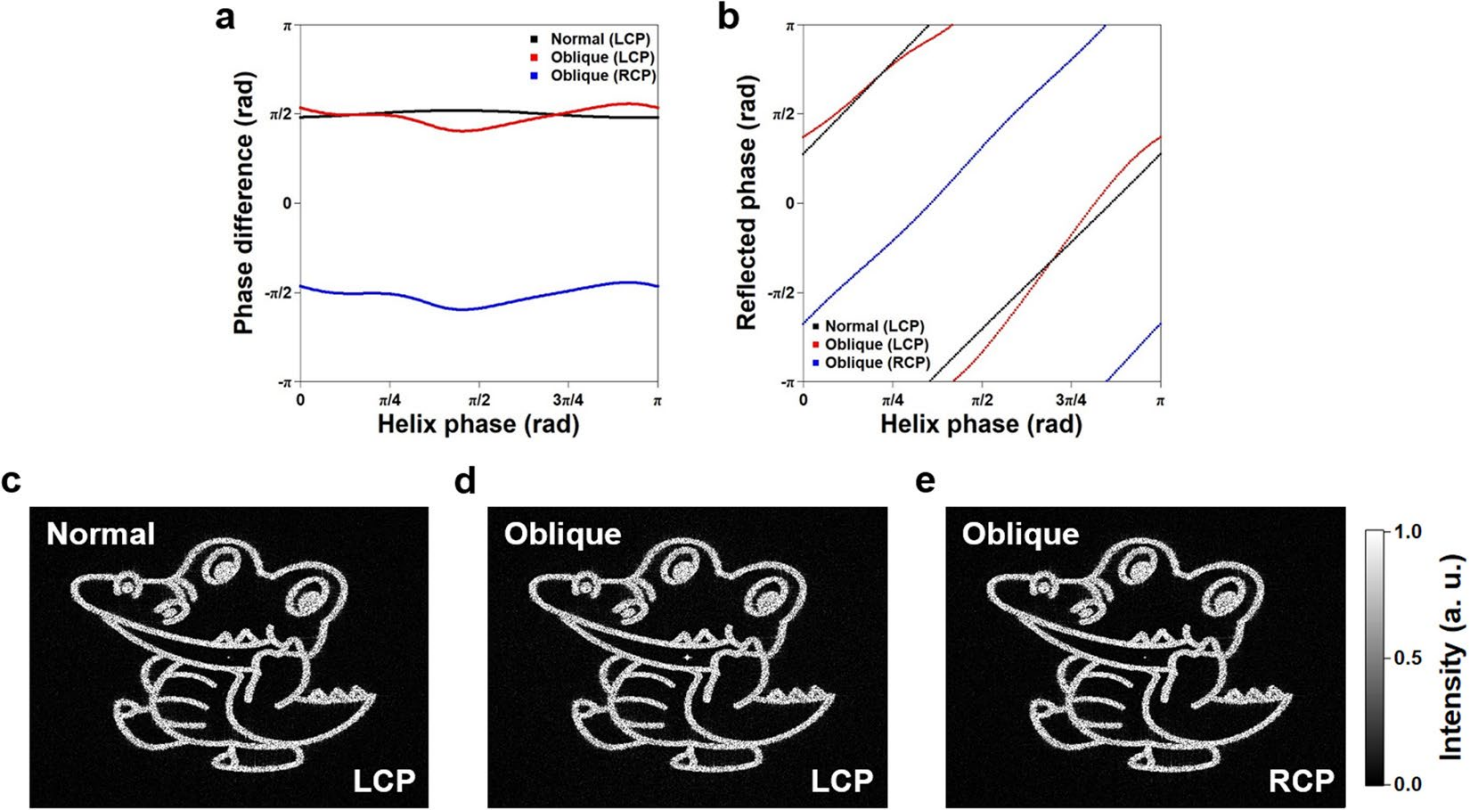
Numerical simulation of the hologram efficiency. (**a**) Phase difference between *p*- and *s*- polarized components of the light reflected from the ChLC for normal (LCP) and oblique (LCP and RCP) incidence. (**b**) Phase of *p*- polarized component reflected from the ChLC depending on incident angle. (**c–e**) Numerically reconstructed holograms for LCP upon normal incidence (**c**), and oblique incident upon LCP (**d**) and RCP (**e**).

The investigated BB hologram was designed assuming a linear relationship between the reflected optical phase and the helix phase^32^. It should be noted that this relationship generally does not hold for largely oblique light incidence. Figure 4a shows the simulated phase difference between *p*- and *s*- polarized components of reflected light depending on the helix phase at normal and oblique incidences (incidence angle: 61° in glass). Results for both CPs are plotted for oblique incidence since both CPs are reflected by almost 100%. For normal incidence, the phase difference between the two polarizations is π/2 (except for a small deviation of 0.04π originating from Fresnel reflection at the substrate surfaces), corresponding to ideal CPs. For oblique incidence, the phase difference deviates from π/2 by at most ±0.15π, and importantly, shows a nonlinear dependence on the helix phase.

The large dependence of reflected polarization on the helix phase implies that the reflected optical phase and helix phase deviates from a linear relationship. While this is detrimental for the hologram efficiency, the effect is not catastrophic, as the measured efficiencies are 75% and 76% for LCP and RCP light, with corresponding diffracted-to-zero order light efficiencies of 91% and 92%. We support this observation by numerically simulating the hologram efficiency for normal and oblique light incidences. Figure 4b shows the optical phase of *p*- polarized component reflected from the ChLC for normal (LCP) and oblique (LCP and RCP) incidence. As expected, a linear relationship is observed for normal incidence, while it is distorted for oblique incidence, with a maximum deviation of 0.14 π. We numerically reconstructed the holograms by the Rayleigh-Sommerfeld diffraction method (see Supplement 1 Section S6 for the simulation method) to investigate this effect on the efficiency. As shown in Fig. 4c–e, the non-linearity in the phase upon oblique incidence causes the 0^th^ order intensity to increase, but its intensity with respect to the total diffracted field is ~1.7% and 0.5% for LCP and RCP light, which is not very high. We believe this to be the reason for the comparable efficiencies obtained at normal and oblique incidences (other than the fact that the hologram design is different). In practice, the offset in the reflected phase can be pre-compensated in the helix phase design by knowing the playback wavelength and incident angle in an optical system, to obtain higher efficiencies.

**Figure 5:**
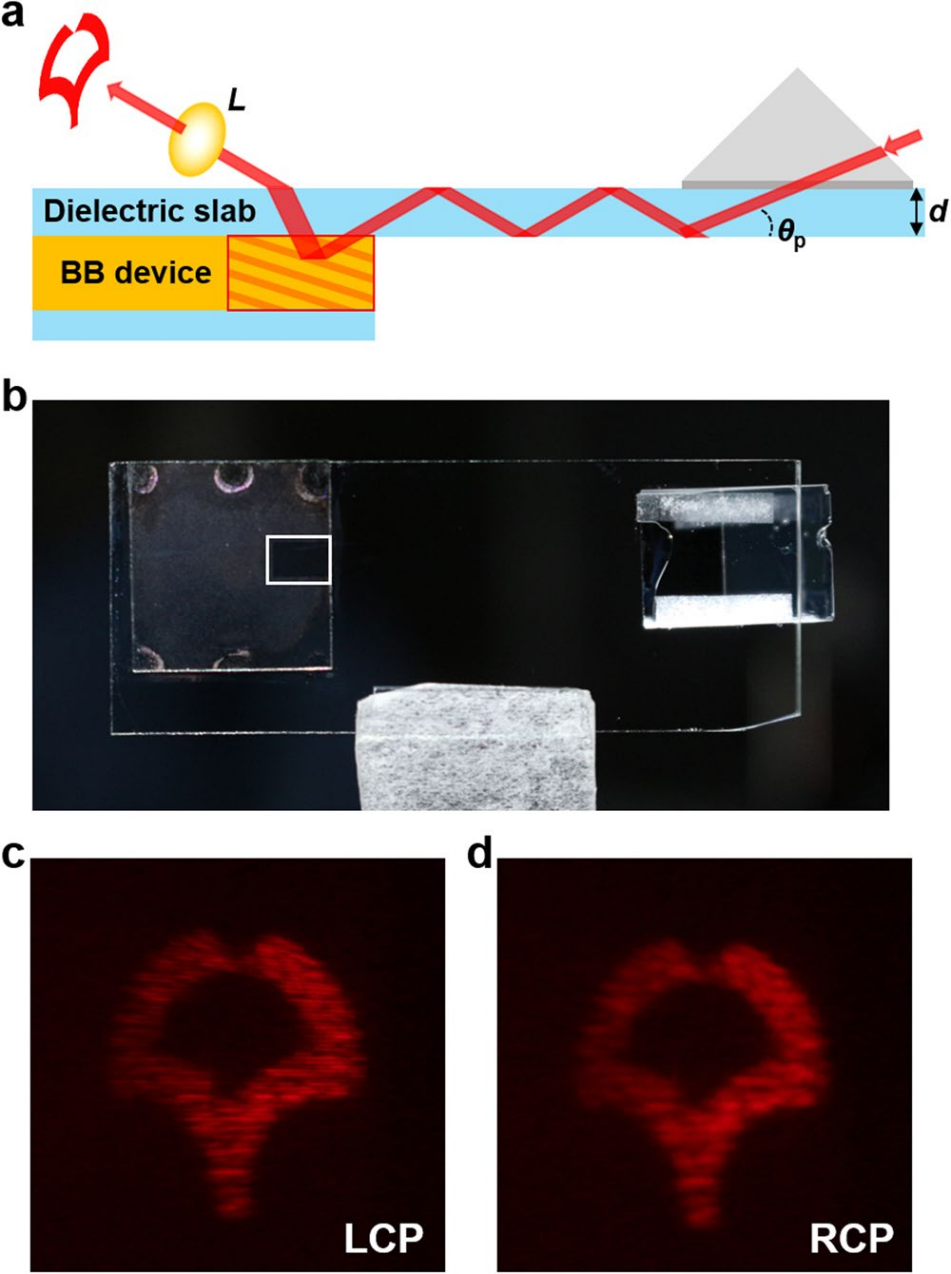
Demonstration of a waveguide BB hologram. (**a**) Schematic diagram of the device. d: thickness of the dielectric slab; *θ*_p_: propagation angle of light; L: lens (*f* = 15 cm). (**b**) Photograph of the device, where the area surrounded by the white square indicates the photo-patterned region with size of ~4 × 2.5 mm^2^. (**c**,**d**) Holographic images out-coupled from the device and observed on a screen for LCP (**c**) and RCP (**d**) light illumination. The playback laser wavelength is 632 ± 5 nm.

As a more realistic demonstration, we show that the proposed device can be combined with a waveguide to enable fully transparent and visible waveguide holography. Figure 5a schematically illustrates the waveguide device in which the BB hologram is attached on a thin dielectric slab (*d* = 0.7 mm) with a refractive index of 1.53. The propagation angle (*θ*_p_) of the waveguided light can be made sufficiently large to cause reflection to occur at visible wavelengths, but because the waveguide is thin and flat, the device appears transparent under normal viewing conditions. To decouple the light out of the waveguide, an off-axis source image is used (see Supplement 1 Section S7 for the source image and helical phase distribution of the waveguide BB hologram). The BB hologram was designed to enable playback at 632 nm using wave-guided light with a propagation angle of *θ*_p_ = 39° (See Supplementary 1 Section S8 for the experimental reflection band of the BB device at the propagation angle of waveguided light).

Figure 5b shows a photograph of the waveguide BB hologram, which clearly shows that the patterned area is completely transparent under the ambient lighting (see Supplement 1 Section S9 for the experimental transmittance of the waveguide BB hologram). The same setup as Fig. 3c was used, but with a different band-pass filter (central wavelength of 632 nm and bandwidth of 10 nm) in the optical path. Figure 5c,d shows the output holographic images for LCP and RCP light incidence, respectively, observed on the screen. Holographic images are clearly visible in free-space, confirming the feasibility of the proposed technology as a fully transparent HOE, but capable to display virtual information when required.

## Discussion

In summary, we have demonstrated the transparent BB hologram: a reflective yet transparent CGH based on a helical anisotropic structure with patterned spatial phase. The helical modulation of the optic axis is key for achieving the transparency, as only a single Fourier component exists in the dielectric constant, and suppresses high-order reflections. In addition, we showed that the device can be played back using visible light, when light is incident from a sufficiently large incidence angle. The device proposed offers the possibility to create advanced security elements where the encoded information is completely transparent under ambient lighting. We also expect that our BB hologram is potentially attractive in augmented reality (AR) applications where auxiliary information is overlapped on the wearer’s field of view. An appropriate pattering process such as interference exposure system^37^ enables a device to possess a large deflection angle over a large area to appropriately overlap virtual information on the wearer’s field of view. In addition, a wide field of view and color uniformity can be achieved by broadening the Bragg reflection band through a ChLC with pitch distribution along the helix axis^42^.

The signal-to-noise ratio (SNR) for the waveguide hologram (Fig. 5c,d) were approximately 25. While we did not attempt to optimize the hologram quality, improvement of hologram quality, which is important for practical applications of the device, should be possible by employing a de-noising phase retrieval method^43,44^ and an alignment patterning process with a higher spatial resolution. Considering that other photonic systems exist that exhibit similar properties to ChLCs, such as helical photonic crystals and metamaterials, we believe that a similar optical effect should be achievable in those systems as well^45^. However, we believe LCs to be one of the most promising candidates to make practical devices, as only a two-dimensional patterning is required to realize the device, and solution-processed films can be made^36,46^. By combining the stimuli-responsive properties of LCs, we foresee the development of HOEs with even more advanced functionalities, such as those that enable dynamic control of properties by an electric field^47,48^, and those that responded to the change in ambient environment^49–51^.

## Materials and methods

### Numerical calculations

The reflection spectra of the ChLC and the standard quarter-wave stack were calculated by Berreman’s 4 × 4 matrix method^40^. The numerical parameters used are summarized in Table 1; the central wavelength of Bragg reflection band for normal incidence was set to ~1150 nm to demonstrate the concept of transparency. Both devices were assumed to be sandwiched by glass substrates with a refractive index of 1.53. Taking the helix axis along the *z*-axis, the ChLC was assumed to exist between z = 0 and 9 µm, with the director oriented along the *x*-axis at *z* = 0. The incident angle was varied in the *z*-*x* plane. The calculations were performed with LCP and RCP light for the ChLC, and linearly polarized light for the quarter-wave stack.

**Table 1:** Numerical parameters used for simulation.

	*n*_e_	*n*_o_	*p*		Thickness of device
ChLC	1.75	1.53	700 nm		9 μm
	*n*_1_	*n*_2_	*d*_1_	*d*_2_	Thickness of device
Quarter-wave stack	2.35	1.53	122 nm	188 nm	9 μm

Parameters used to calculate the reflection spectra of the ChLC and the quarter-wave stack. The values of *n*_o_ and *n*_e_ is the ordinary and extraordinary refractive indices, and *p* is the helical pitch. The values of *n*_1_ and *n*_2_ are the refractive indices of two dielectric materials, *d*_1_ and *d*_2_ are the corresponding thickness of each layer.

The dependence of optical phase of *p*- and *s*- polarized components reflected from the BB hologram on the helix phase (Fig. 4a,b) was also calculated by the 4 × 4 matrix method^40^. The helix phase was defined as the azimuth angle of the LC director from the *x*-axis at *z* = 0. Simulations and analyses were performed using home-built code in a commercial data analysis software (Wavemetrics, Igor Pro 7).

### Materials

For the experiments in Figs. 2 and 3, the left-handed ChLC material was prepared by mixing a LC mixture (Merck, MLC-2140) with a left-handed chiral dopant (HCCH, S-5011) at a weight ratio of 98.8:1.2. For the waveguide experiment (Fig. 5), the two materials were mixed at a slightly different weight ratio of 97.7:1.3. In all experiments, the ChLCs were injected into the patterned cells in the isotropic phase (100 °C) and cooled to room temperature at a rate of 0.3 °C/min. The reflection spectrum was measured using a home-built microscope setup equipped with a ×4 objective and an optical fiber with 1 mm diameter coupled to a spectrometer (Hamamatsu, PMA-12). The transmission spectrum was measured on a polarizing optical microscope (Nikon, LV-100-POL) equipped with a ×10 objective and a 1 mm-thick optical fiber coupled to a spectrometer (Hamamatsu, PMA-11).

### Device fabrication

For the experiments in Figs. 2 and 3, the sandwiched cell was prepared from one substrate covered with a photoalignment material (DIC, LIA-03) and another coated with polyimide (JSR, AL1254) and rubbed unidirectionally. An azo-benzene based photoalignment material was used which sets the orientational easy axis of the LC in the direction perpendicular to the incident polarization. The two substrates were assembled to a gap of 9 µm. For fabrication of the waveguide BB hologram (Fig. 5), the photoalignment material was coated on two glass substrates with sizes of 5 × 2 cm^2^ (waveguide) and 1.5 × 1.5 cm^2^. The two substrates were sandwiched to a cell-gap of 9 µm, placing the smaller substrate at one end of the larger substrate. A glass prism made from BK-7 was attached at the other end to couple light into the waveguide for playback.

The patterning process was performed using a maskless projection system^30^ comprising an electronic mask (EPSON, ELP-820), a band-pass filter (central wavelength of 436 nm with bandwidth of 10 nm), and polarization and projection optics. The system irradiates light on the sample with a controlled linear polarization angle over an area controlled by the electronic mask. The pixel resolution of the mask was 1024 × 768, with the theoretical size of a single pixel being approximately 1.33 × 1.33 μm^2^ on the sample. For the waveguide BB hologram, a projection lens with a shorter focal length was used to achieve a theoretical pixel size of approximately 0.33 × 0.33 μm^2^. The polarization angle was rotated at 10° increments, corresponding to 18 phase levels in the CGH. The light dosage for patterning was 0.5 μJ/pixel for the normal device and 0.1 μJ/pixel for the waveguide device.

### Evaluation of signal-to-noise ratio (SNR)

The SNR is defined as the ratio of the maximum intensity of the holographic image to the standard deviation of the background noise^13^. The SNR was determined using an area that corresponds to 100 × 100 pixels of the target image, centered around the Osaka University logo (5.6 × 7.5 cm^2^ on the screen located at 30 cm from sample). The maximum intensity of the projected logo was divided by the standard deviation of the intensity in the areas corresponding the pixels outside the logo, giving values of 24.0 and 25.8 for the waveguide hologram played back using LCP and RCP light.

## Supplementary information

Supplementary information.

Supplementary information2.
