# Supplementary material for: Bragg-Berry flat reflectors for transparent computer-generated holograms and waveguide holography with visible color playback capability
Source: Sci Rep. 2020 May 18;10:8201. doi: 10.1038/s41598-020-65102-0 (PMC7235227; doi:10.1038/s41598-020-65102-0)
Supplement: Supplementary file 2 — Supplementary information2. [file 41598_2020_65102_MOESM2_ESM.docx]

Supplementary information for

**Bragg-Berry flat reflectors for transparent computer-generated holograms and waveguide holography with visible playback capability**

SeongYong Cho^1^, Masaru Ono^1^, Hiroyuki Yoshida^1,2*^ and Masanori Ozaki^1^

1. Division of Electrical, Electronic and Information Engineering, Osaka University, 2-1 Yamadaoka, Suita, Osaka 565-0871, Japan.

2. Precursory Research for Embryonic Science and Technology (PRESTO), Japan Science and Technology Agency (JST), 4-1-8 Honcho, Kawaguchi, Saitama 332-0012, Japan

*Correspondence to: yoshida@eei.eng.osaka-u.ac.jp

Tel: (81)6 – 6879 – 7759

Fax: (81)6 – 6879 – 4838

**S1. Phase retrieval algorithm.**

The optical phase distribution for the hologram design was retrieved by the Gerchberg-Saxton (G-S) algorithm [1]. We first prepared the source image and a random optical phase distribution in a Fourier plane with a projection area of 512 × 384 pixels. After combining the source image with the random phase distribution, it was inverse-Fourier-transformed to get an optical information at an objective plane. The amplitude of the obtained optical information was replaced with a planar amplitude, and then was Fourier-transformed to reproduce the diffraction pattern in the Fourier plane. The diffraction pattern was substituted with the desired diffraction pattern (source image). This algorithm was repeated 100 times to achieve that the zero mean normalized cross-correlation between the source image and the reconstructed image exceed 0.98. The final optical phase information was converted to the helical phase distribution by multiplying a factor of 0.5. The total calculation time took 30s in whole procedure.

**S2. Broadening the accessible incident angle on the transparent device.**

Figure S1a schematically illustrates the ChLC cell attached to a triangular prism to increase the accessible angles of light incidence. Refraction at the prism surface increases the increases the incident angle in the ChLC, *θ*_3_, to be given by the following expression:

$\theta_{3}=\sin^{-1}\left[\frac{n_{\text{p}}}{n_{\text{g}}}\sin\left\{45^{^{\circ}}+\sin^{-1}\left(\frac{1}{n_{\text{p}}}\sin\theta_{1}\right)\right\}\right]$. (S1)

In Eq. S1, *n*_p_ and *n*_g_ are the refractive indices of the prism and glass substrate, and *θ*_1_ is the incident angle of light into the prism. In experiment, the prism was attached onto the cell using an index matching oil (Olympus, IMMOIL-F30CC, note that it has no effect on *θ*_3_), and *n*_p_ = 1.5148 and *n*_g_ = 1.53. Fig. S1b shows the dependence of *θ*_3_ on *θ*_1_, where as a reference, the angles for the device without the prism also plotted (given by *θ*_3_ = sin^-1^(1/*n*_g_ sin(*θ*_1_)). The maximum incident angle accessible in the ChLC is increased from 3° to 81°. Note that the triangle prism was employed for facile demonstration of visible light playback and for practical applications, other means can also be used, as long as the accessible angle inside the ChLC is increased.





**Fig. S1. Increasing the accessible angle by a prism.** **(a)** The schematic of the prism-coupled ChLC cell. **(b)** Accessible incident angle with/without the prism on the cell.

**S3. Dependence of the ChLC reflection spectrum on the incident angle.**

The transparent ChLC material was filled into a sandwich cell with planar alignment layers to prepare a uniformly aligned sample. The cell was then attached to a triangular prism to increase the accessible incident angle. The polarization of the super-continuum light source was controlled by using a polarizer and a quarter-wave plate, and illuminated on the sample to measure the reflection band of the ChLC at various incident angles. The sample was placed on a rotatable stage, and light reflected at the angle twice that of sample rotation was collected using a bundled fiber (diameter: 1 mm) and measured using a spectrometer (Hamamatsu, PMA-11). Figure S2 shows the experimentally obtained angular dependence of the reflection spectrum in the visible light region. For LCP incidence, Bragg reflection appears at ~800 nm at 45°, and blue-shifts to ~490 nm at 68°. For RCP incidence, Bragg reflection appears at approximately the same wavelength and gradually widens with increasing incident angle. These results are in agreement with the simulation presented in Fig. 1 of the main text.


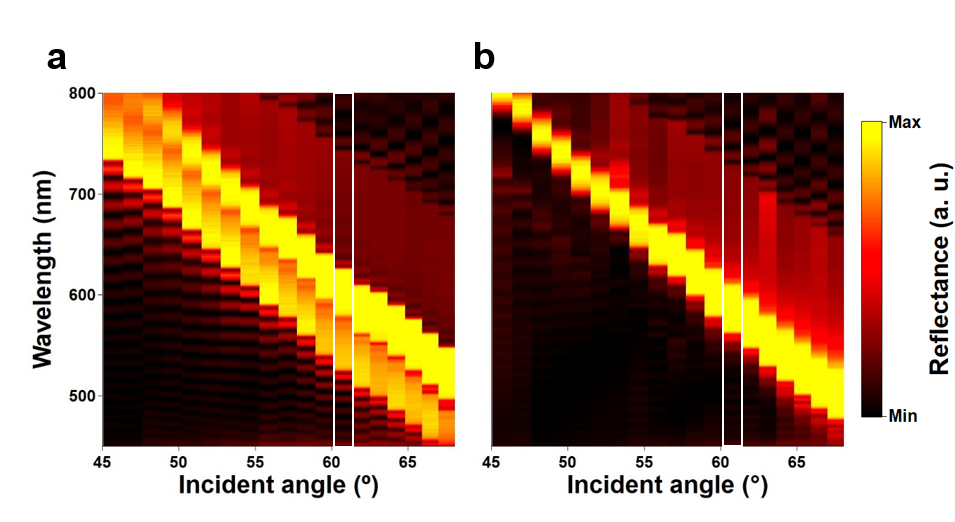


**Fig. S2 Incident angle dependence of the reflection spectrum for a ChLC at largely oblique incident angles.** **(a,b)** Reflection spectra of a left-handed ChLC at various angles of incidence for LCP **(a)** and RCP **(b)**, respectively. The region surrounded by the two white lines indicate the approximate incident angles that were accessed in experiment, when the BB hologram was played back at 580 ± 5 nm.

**S4. Physical mechanism of polarization-independent reflection from the ChLC.**

It is well known that ChLCs show perfect CP selectivity for light propagating along the helix axis, because of the sinusoidal dielectric tensor distribution schematically illustrated in Fig. S3a. However, for large angles of incidence, they exhibit a blue-shifted Bragg reflection band that is independent of the incident polarization, which is referred to as the total reflection band [2, 3]. This is because the dielectric tensor distribution becomes shortened and deviates from a complete sinusoidal wave, as schematically illustrated in Fig. S3b. As the angle of incidence increases, this phenomenon becomes more prominent, resulting in the increase of total reflectance and the width of total reflection band.


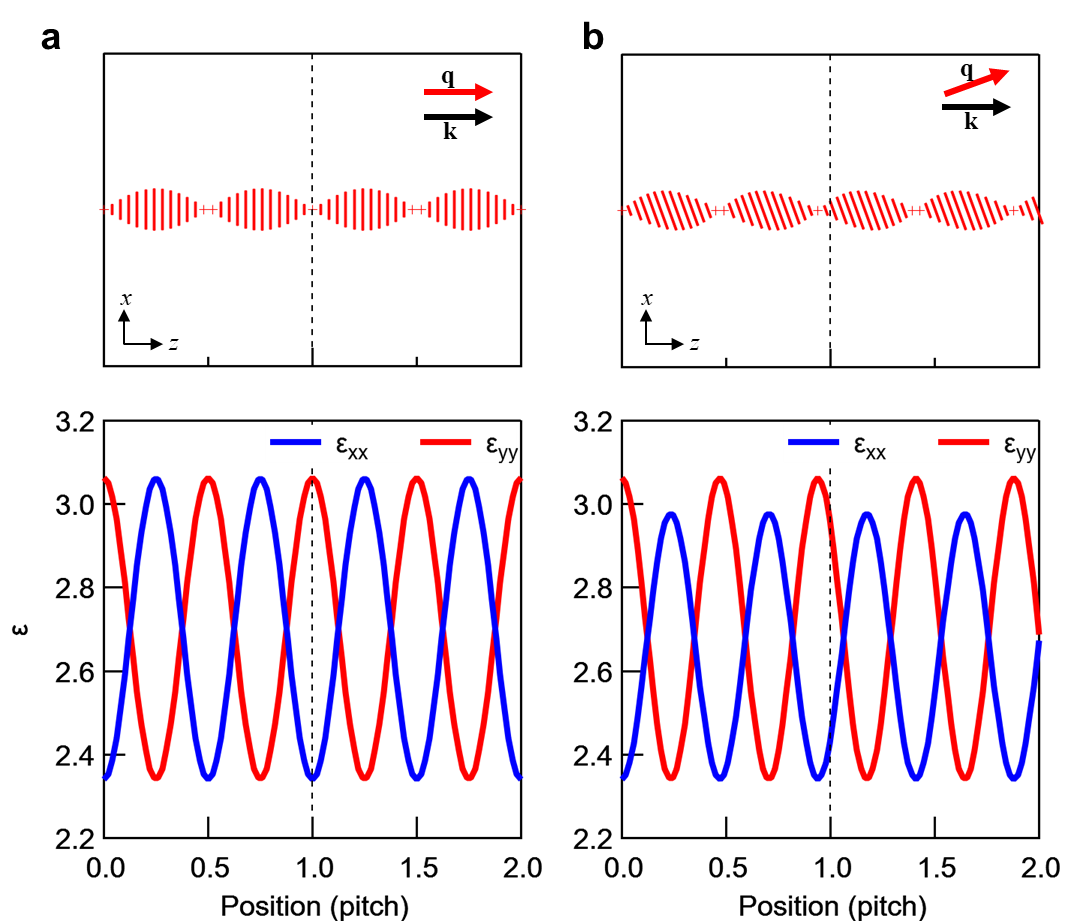


**Fig. S3 Dielectric tensor distribution of a ChLC depending on the propagation direction of light. (a)** Schematic illustration of the director and dielectric tensor distributions for light propagating along the ChLC helix axis. **q** and **k** indicate the helical axis and light propagation directions, respectively. **(b)** The director and dielectric tensor distributions for light propagating at 20° to the helix axis.

**S5. Hologram design and helix phase distribution for oblique incidence experiments.**

To design helix phase distribution for oblique incidence, the source image was enlarged by 3 times and centered around the zero-order spot as shown in Fig. S4a. Because the hologram is designed for playback by an obliquely incident light, the width of the source image was reduced by the value of cos *θ*, where *θ* is the angle of incident light. The optical phase distribution was obtained by the Gerchberg-Saxton algorithm as described in the main text [1]. The final optical phase distribution was divided by two to obtain the helix phase distribution as shown in Fig. S4b. The helix phase distribution was patterned on the device at a phase step of 10°. As discussed in the main text, the design here assumes a linear relationship between optical phase and the helix phase, which does not hold upon oblique light incidence. The offset in phase can be pro-compensated by knowing the accurate relationship between the reflected optical phase and the helix phase as shown in Fig. 4b of the main text.





**Fig. S4 Design of hologram for playback by obliquely light incidence.** **(a)** Source image of the Osaka University mascot Dr. Wani. **(b)** Calculated helix phase distribution corresponding to the source image.

**S6. Simulation of light propagation based on Rayleigh-Sommerfeld diffraction.**

The Rayleigh-Sommerfeld diffraction (R-S) is the most useful for the simulation of our device because it allows the calculation of diffracted fields propagating to large angular direction [4, 5]. The equation of R-S diffraction is expressed mathematically as,

$\begin{matrix} U\left(x,y,z\right) = \iint_{A} U\left(x_{0},y_{0},0\right)\frac{\exp(ikr)}{2\pi r}\left(\frac{1}{r}-ik\right)\frac{\Delta z}{r}dx_{0}dy_{0} \\ \\ r = \sqrt{\left(x-x_{0}\right)^{2}+\left(y-y_{0}\right)^{2}+\Delta z^{2}} \end{matrix}$, (S2)

where *U*(*x*_0_, *y*_0_, 0) is the electric field distribution in the aperture plane, *k* = 2π/*λ* is the wavevector, *λ* is the wavelength of light, and Δ*z* is the distance between the aperture plane and the objective plane. The integral of Eq. S2 can be calculated by direct numerical integration [4] with discrete *U*(*x*_0_, *y*_0_, 0) sampled in N × M pixels, as a Riemann sum,

$\begin{matrix} U\left(x_{m},y_{n},z\right) = \sum_{i=1}^{N} \sum_{j=1}^{M} U(x_{0i},y_{0j},0)\frac{\exp(ikr)}{2\pi r}\left(\frac{1}{r}-ik\right)\frac{\Delta z}{r}\Delta x_{0}\Delta y_{0} \\ \\ r = \sqrt{\left(x_{n}-x_{0i}\right)^{2}+\left(y_{m}-y_{0j}\right)^{2}+\Delta z^{2}} \end{matrix}$. (S3)

The discrete *U*(*x*_0_, *y*_0_, 0) was prepared by converting the helix phase distribution [Fig. S4b] into the reflected phase distribution based on the relationship described in Fig. 4b of main text. Figures S5 shows the reflected phase distribution at incident angle of 0° and 61° from the BB hologram device. The electric field distribution in the aperture plane was prepared by combining the reflected phase distribution with a uniform amplitude. The calculation was conducted using the values of the pixel size (Δ*x*_0_ = Δ*y*_0_ = 2.7 μm), the number of sampling grid (N = 512, M = 384), and the distance (Δ*z* = 30 cm) achieved in experiment.


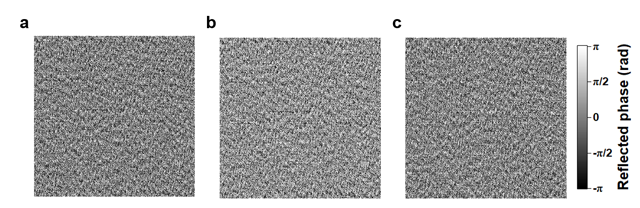


Fig. S5. Reflected optical phase distribution for normal and oblique light incidence. (a) Reflected optical phase distribution for the BB hologram of Fig. S4, calculated by LCP light under normal incidence, using the optical-to-helix phase relation of Fig. 4b in main text. (b,c) Reflected optical phase distribution for the BB hologram calculated for LCP (b) and RCP (c) light under oblique incidence (61º), obtained using the optical-to-helix phase relationship of Fig. 4b in main text.

**S7. Design of the waveguide BB hologram.**

Figure S6a shows the design of the hologram used in the waveguide device (Fig. 5 in main text), where the holographic image (Osaka University logo) is designed to appear 190 pixels away from the zero-order spot in the horizontal direction. The center of the hologram is deflected at an angle given by the following equation [4],

$\theta_{d}=\tan^{-1}\left(\frac{N_{a}\lambda}{nL_{x}}\right)$, (S4)

where *θ_d_* is the deflection angle, *N_a_* is the pixel number from zero-order spot to the center of holographic image, *λ* is the wavelength of light, *n* is the refractive index of light-propagating space, and *L*_x_ is the horizontal length of the CGH. Based on values achieved in experiment where *N_a_* is 190 pixels, *λ* is 632 nm, *n* is 1.53 (waveguide slab), and *L*_x_ is 338 μm, the holographic image is reconstructed with a deflection angle of 13.1º in the waveguide layer. Deflection of waveguided light by this angle makes the total internal reflection condition to be violated, resulting in out-coupling of the hologram. The helical phase distribution shown in Fig. S6b was obtained by the Gerchberg-Saxton algorithm [1] as described in Section S5. The helix phase distribution was photo-patterned on the device with a phase step of 10°.


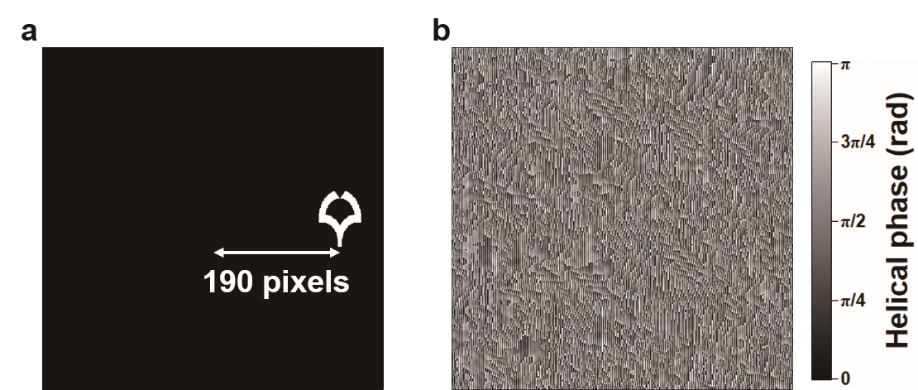


**Fig. S6 Design of hologram for waveguide BB hologram.** **(a)** Source image, where the Osaka University logo is placed 190 pixels away from the zero-order spot. **(b)** Obtained helix phase distribution corresponding to the source image.

**S8. Reflection spectrum of waveguide BB hologram upon oblique incidence.**

In the waveguide BB hologram device in Fig. 5a of the main text, light is guided through the dielectric slab at a propagation angle of 39°, making the wave-guided light to be incident on the BB hologram at an angle of 51° from the boundary normal of the device. Figure S7 shows a reflection spectrum upon LCP and RCP illumination at a given incident angle, which is obtained through the same method described in Section S2. The total reflection band appears around 630 nm with a bandwidth of approximately 25 nm, enabling the encoded phase information to be played back using a light with wavelength of 632 nm ± 5 nm.


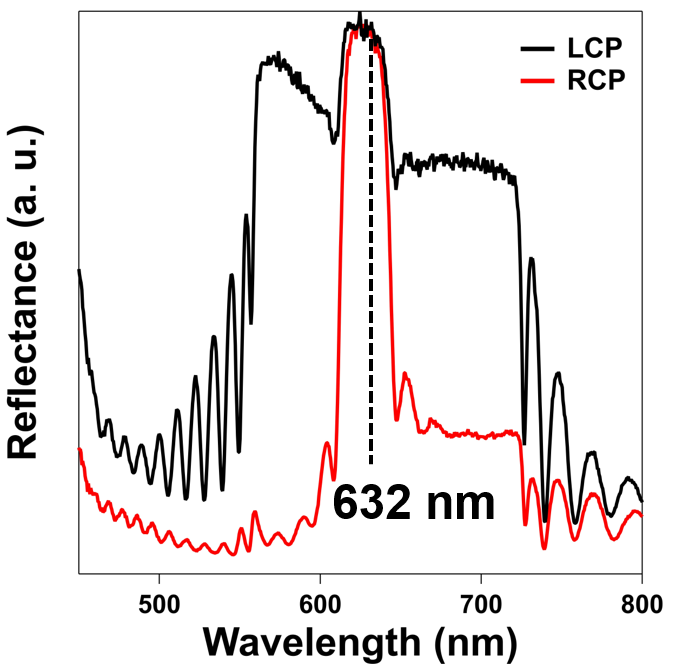


**Fig. S7 Reflection spectra of waveguide BB hologram for LCP and RCP light illumination at an incident angle of 51°.**

**S9. Transmission spectrum of the waveguide BB hologram.**

The transmission spectrum was obtained on a polarizing optical microscope (Nikon, LV-100-POL) equipped with a × 10 objective and a 1 mm-thick optical fiber coupled to a spectrometer (Hamamatsu, PMA-11). Figure S8 shows the transmission spectra of the waveguide BB hologram in the visible range. No high-order reflections are observed in visible light region, with average transmittance of 88%. There exists a slight reduction in the transmittance compared to the normal BB hologram due to the imperfection of the photo-patterning process, which causes unwanted light scattering. Improvement in the transmittance can be achieved by employing a patterning process with higher spatial resolution, as well as placing an antireflection coating on the device.





**Fig. S8 Transmission spectra of waveguide BB hologram in the visible light region.**

**Supplementary Movie 1. Visible playback of transparent hologram device.** Two identical BB holograms are placed side by side, with a triangular prism placed only on the right device. Because the prism widens the angle of incidence, the red laser beam becomes Bragg-reflected, leading to playback of the hologram encoded in the spatial phase distribution of the ChLC. The device without the prism only shows a single spot on the screen attributed to Fresnel reflection.

**References**

1. Gerchberg, R. W. & Saxton, W. O. Practical algorithm for determination of phase from image and diffraction plane pictures. *Optik* **35**, 237–246 (1972).

2. Ozaki, R. & Moritake, H. Wavelength and bandwidth tunable photonic stopband of ferroelectric liquid crystals. *Opt. Express* **20**, 6191-6196. (2012).

3. Takezoe, H., Ouchi, Y., Hara, M., Fukuda, A. & Kuze, E. Experimental studies on refection spectra in monodomain cholesteric liquid crystal cells: Total reflection, subsidiary oscillation and its beat or swell structure. *Jap. J. Appl. Phys.* **22**, 1080-1091 (1983).

4. Goodman, J. W. Introduction to Fourier Optics (McGraw-Hill, 1996).

5. Shen, F. & Wang, A. Fast-Fourier-transform based numerical integration method for the Rayleigh–Sommerfeld diffraction formula. *Appl. Opt.* **45**, 1102–1110 (2006).
